# Brewpitopes: a pipeline to refine B-cell epitope predictions during public health emergencies

**DOI:** 10.3389/fimmu.2023.1278534

**Published:** 2023-12-06

**Authors:** Roc Farriol-Duran, Ruben López-Aladid, Eduard Porta-Pardo, Antoni Torres, Laia Fernández-Barat

**Affiliations:** ^1^ Barcelona Supercomputing Center (BSC), Barcelona, Spain; ^2^ CELLEX Research Laboratories, CibeRes (Centro de Investigación Biomédica en Red de Enfermedades Respiratorias, Institut d’Investigacions Biomèdiques August Pi i Sunyer (IDIBAPS), Barcelona, Spain; ^3^ Pneumology Department, Hospital Clínic, Barcelona, Spain; ^4^ Josep Carreras Leukaemia Research Institute (IJC), Badalona, Spain

**Keywords:** bioinformatics and computational biology, immunology and infectious diseases, vaccine development, antibody therapeutics, epitope prediction and antigenicity prediction

## Abstract

The application of B-cell epitope identification to develop therapeutic antibodies and vaccine candidates is well established. However, the validation of epitopes is time-consuming and resource-intensive. To alleviate this, in recent years, multiple computational predictors have been developed in the immunoinformatics community. Brewpitopes is a pipeline that curates bioinformatic B-cell epitope predictions obtained by integrating different state-of-the-art tools. We used additional computational predictors to account for subcellular location, glycosylation status, and surface accessibility of the predicted epitopes. The implementation of these sets of rational filters optimizes *in vivo* antibody recognition properties of the candidate epitopes. To validate Brewpitopes, we performed a proteome-wide analysis of SARS-CoV-2 with a particular focus on S protein and its variants of concern. In the S protein, we obtained a fivefold enrichment in terms of predicted neutralization versus the epitopes identified by individual tools. We analyzed epitope landscape changes caused by mutations in the S protein of new viral variants that were linked to observed immune escape evidence in specific strains. In addition, we identified a set of epitopes with neutralizing potential in four SARS-CoV-2 proteins (R1AB, R1A, AP3A, and ORF9C). These epitopes and antigenic proteins are conserved targets for viral neutralization studies. In summary, Brewpitopes is a powerful pipeline that refines B-cell epitope bioinformatic predictions during public health emergencies in a high-throughput capacity to facilitate the optimization of experimental validation of therapeutic antibodies and candidate vaccines.

## Introduction

Neutralizing antibodies play a major role in the adaptive immune response against pathogens (1). Hence, the prediction of the protein regions driving pathogen neutralization is key to guide the understanding of their mechanism of action (1). These protein regions, termed neutralizing B-cell epitopes, have the potential to spread through the entire proteome of the target pathogen. Such a wide distribution requires high-throughput techniques to unravel the full epitope landscape. In this context, the bioinformatic prediction of B-cell epitopes has become a necessary exploration to prioritize which candidates should be selected for experimental validation (**Table 1**). For instance, in the race against the SARS-CoV-2 pandemic, accurate bioinformatic B-cell epitope predictors significantly contributed to the success of COVID-19 preventive and therapeutic strategies (22) (**Table 1**). For this reason, many groups dedicated their efforts to the identification of SARS-CoV-2 antibody binding regions using different bioinformatic approaches as a first step to later characterize neutralizing antibodies or to design immunogens for vaccines (**Table 1**) (22, 23).

**Table 1:** Biophysical features included in state-of-the-art B-cell epitope predictors and the strategies followed for epitope landscape determination during early SARS-CoV-2 pandemics.

A															
Method	Type of Epitope		Subcellular Location		Glycosylation		Surface Accessibility		Based on		Comment			Reference	
Bepipred 2.0	Linear		No		No		No		Antibody–antigen structures		Buried residues are predicted within epitopes			(2)	
ABCpred	Linear		No		No		No		BCIPEP		High numbers of epitopes due to different window lengths			(3, 4)	
EpitopeVec	Linear		No		No		No		IEDB+ BCIPEP		–			(4–6)	
SVMTRIP	Linear		No		No		No		IEDB		–			(6, 7)	
Discotope 2.0	Conformational		No		No		No		Antibody–antigen structures		–			(6, 8)	
SeRenDIP-CE	Conformational		No		No		No		SAbDab		–			(9, 10)	
SEPPA3.0	Conformational		Yes		Yes		No		Antibody–antigen structures		Predefined subcellular location. Does not account for surface accessibility or O-glycosylations.			(11)	
Ellipro	Conformational		No		No		No		Antigen structures		–			(12)	
Epitope3D	Conformational		No		No		No		Antibody–antigen structures		–			(13)	
Brewpitopes	Linear + Conformational		Yes		Yes		Yes		Antibody–antigen structures		–			–	
B.															
Authors		Type of Epitope		Epitope Predictor		Subcellular Location		Glycosylation		Surface Accessibility		Based on	Comment		Reference
Almoftl et al.		Linear+ Conformational		IEDB		No		No		No		IEDB			(14)
EzaJ et al.		Linear+ Conformational		IEDB		No		No		No		Molecular dynamics			(4)
Sikora et al.		Conformational		–		No		Yes		No		Homology modeling			(15)
Khare et al.		Conformational		–		No		No		Yes		–	Integrates sequence conservation and functional domains.		(16)
Smith et al.		Linear		–		No		Yes		Yes		Epitope mapping			(17)
Li et al.		Linear		–		No		No		No		Epitope mapping			(18)
Schwarz et al.		Linear		–		No		No		No		Homology modeling			(19)
Grifoni et al.		Linear		IEDB		No		No		Yes		Bacterial display libraries			(20)
Haynes et al.		Conformational		SERA		No		No		No		Ab–Antigen structures			(21)
Farriol-Duran et al.		Linear+ Conformational		Multiple		Yes		Yes		Yes					

(A) Comparison of the state-of-the-art B-cell epitope predictors. Classification of the different tools according to the inclusion of subcellular location, glycosylation status, and surface accessibility. Training datasets are annotated in the “Based on” column.

(B) Comparison of the B-cell epitope studies of SARS-CoV-2. Collection of B-cell epitope prediction approaches analyzing the epitope landscape of SARS-CoV-2. Strategies were classified according to the aforementioned biophysical constraints. B-cell epitope predictors and validation techniques are annotated in the “Based on” column.

B-cell epitope predictors recommended by the Immune Epitope Database (IEDB) (24) such as Bepipred (2), or Discotope (8), and other existing SOTA methods (**Table 1**) (5, 7, 9–13) are tools able to identify candidate continuous and discontinuous B-cell epitopes in a minute scale. However, even state-of-the-art B-cell epitope prediction tools frequently output lists of predicted epitopes that are excessively large to validate experimentally (25). Moreover, many of the predicted epitopes will not necessarily function *in vivo* (25). Hence, the development of new predictive tools that will refine the available computational B-cell epitope predictions is a priority. Such tools will provide a rapid and accurate reaction in case of emergency situations such as the COVID-19 pandemic or the appearance of new variants of concern (VOCs) that escape the immune response of vaccinated subjects (3, 26).

To this end, we have designed Brewpitopes, a new predictive pipeline that integrates additional important features of known epitopes, such as glycosylation or structural accessibility using specific computational methods. To curate B-cell epitope predictions for neutralizing antibody recognition, Brewpitopes outputs curated lists of refined epitopes with an increased likelihood to be functional *in vivo*. To validate Brewpitopes, the pipeline was implemented to predict B-cell epitopes in antibody binding regions on the entire the proteome of SARS-CoV-2, with a special focus on the S protein and its VOCs.

## Materials and methods

All three-dimensional protein figures have been generated with PyMol 2.5 and Chimera X. All statistical analyses have been performed using R statistical software (R version 3.6.3). All data and software can be obtained from public sources for academic use.

### Dataset curation

The SARS-CoV-2 proteome in UniprotKB consists of 16 reviewed proteins (27). We used the corresponding FASTA sequences as starting data for linear epitope predictions. To perform structural epitope predictions, when available, we obtained the PDB structures from the Protein Data Bank database selecting the structures with the best resolution and more protein sequence coverage (28). For those proteins with no available structure in PDB, we used Alphafold2.0 (29) or Modeller (30) to model their 3D structure.

### Linear epitope predictions

To predict linear epitopes on protein sequences, we used ABCpred (31) and Bepipred 2.0 (2). We used ABCpred (31), an artificial neural network trained on B-cell epitopes from the Bcipep database (32), to predict linear epitopes given a FASTA sequence. The identification threshold was set to 0.5 as indicated by default (accuracy 65.9%) and all the window lengths were used for prediction (10–20mers). Additionally, we kept the overlapping filter on. To further augment the specificity of the predictions, we increased the ABCpred score to 0.8.

In addition, we used Bepipred 2.0 (2), a random forest algorithm trained on epitopes annotated from antibody–antigen complexes, as a second source to predict linear epitopes. The epitope identification threshold was set to ≥0.55 leading to a specificity of 0.81 and a sensitivity of 0.29 (32).

### Structural epitope predictions

We used PDBrenum (33) to map the PDB residue numbers to their original positions at the UniprotKB FASTA sequence. The reason behind this step was that factors such as the inclusion of mutations to stabilize the crystal may lead to discordances between the residue numbers in the PDB and FASTA sequence from the same protein.

In order to model those SARS-CoV-2 proteins with missing structures in PDB, we used Alphafold 2.0 (29). We then refined the models by restraining our analysis to those regions with a pDLLT threshold of 0.7 to only assess highly confident regions. The proteins that required Alphafold modeling were M, NS6, ORF9C, ORF3D, ORF3C, NS7B, and ORF3B.

To predict conformational or structural B-cell epitopes, we used Discotope 2.0, a method based on surface accessibility and a novel epitope propensity score (8). The epitope identification threshold was set to −3.7, as specified by default, which determined a sensitivity of 0.47 and a specificity of 0.75.

### Epitope extraction and integration

Bepipred 2.0 (2), ABCpred (31), and Discotope 2.0 (8) predictions resulted in different tabular outputs. To extract and curate the predicted epitopes, we created a suite of computational tools in R statistical programming language and Python, available at https://github.com/rocfd/brewpitopes.

### Subcellular location predictions

When publicly available, the protein topology information was retrieved from the subcellular location section in UniprotKB (27). For those proteins with unavailable topology, we predicted their extracellular regions using Constrained Consensus TOPology prediction (CCTOP) (6), a consensus method based on the integration of HMMTOP (34), Membrain (35), Memsat-SVM (36), Octopus (37), Philius (38), Phobius (39), Pro and Prodiv (40), Scampi (41), and TMHMM (42). The.xml output of CCTOP was parsed using an in-house R script (xml_parser.R) and then the extracted topology served as reference to select epitopes located in extracellular regions using the script Epitopology.R.

### Glycosylation predictions

To investigate *in silico* which residues would be glycosylated, we used NetNGlyc 1.0 (43) for N-glycosylation and Net-O-Glyc 4.0 (44) for O-glycosylations. NetNglyc uses an artificial neural network to examine the sequences of human proteins in the context of Asn-Xaa-Ser/Thr sequons. NetOglyc produces neural network predictions of mucin type GalNAc O-glycosylation sites in mammalian proteins. We parsed the corresponding outputs using tailored R scripts and then we extracted the glycosylated positions to filter out those epitopes containing glycosylated residues using Epiglycan.py.

### Accessibility predictions

To predict the accessibility of epitopes within their parental protein structure, we computed the relative solvent accessibility (RSA) values using ICM browser from Molsoft (45). We used an in-house IEC browser script (Compute_ASA.icm) to compute RSA and we considered buried those residues with RSA threshold less than 0.20. Then, the ICM-browser output was parsed to extract the buried positions, which then served as a filter to discard epitopes containing inaccessible or buried residues using Episurf.py.

### Variants of concern analysis

The mutations accumulated by the VOCs Alpha, Beta, Delta, Gamma, and Omicron in the S protein were obtained from the CoVariants webpage (4), which is empowered by GISAID data (46). A fasta sequence embedding each variant’s mutations was generated using fasta_mutator.R.

## Results

### Brewpitopes, a pipeline to curate B-cell epitope predictions based on determinant features for *in vivo* antibody recognition

While there are some tools available to predict the presence of B-cell epitopes in a protein sequence or structure, these tools are mainly based on machine learning methods trained with experimentally validated epitopes (**Table 1**). However, these methods sometimes do not account for other factors that might affect the antigenicity or the potential of a protein region to be recognized specifically by antibodies.

**Figure 1:**
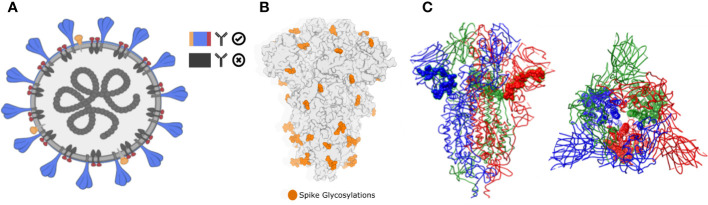
Biophysical constraints for *in vivo* antibody recognition. **(A)** Recognition of extracellular or extra-viral protein regions. Neutralizing antibodies only inspect the external surface of viral particles. Therefore, predicted epitopes located in intracellular or transmembrane epitopes will not be recognized. In Brewpitopes, we used protein topology-annotated information and topology predictors to assess the subcellular location of the target protein regions with predicted epitopes. Exclusively, candidates located on extracellular protein regions were selected. **(B)** Glycosylation coverage prevents *in vivo* antibody recognition of neutralizing epitopes. Predicted epitopes that contain glycosylation motifs are likely covered by glycans supporting the selection of predicted epitopes without glycosylated residues. In Brewpitopes, we predicted the glycosylation profiles of target proteins using Net-N-glyc and Net-O-glyc for N- and O-glycosylations, respectively. Only predicted epitopes without glycosylated residues pass this filter. **(C)** Epitope accessibility on parental protein surface. Predicted epitopes that contain buried residues will be less accessible for *in vivo* antibody recognition. Left: structure of S protein of SARS-CoV-2 highlighting a fully accessible predicted epitope. Right: structure of the S protein displaying a highly buried predicted epitope. In Brewpitopes, to assess epitope accessibility, we calculated the Residue Solvent Accessibility (RSA) of the predicted epitope sequences using crystal or structural models. Once predicted, fully accessible epitopes (all residues RSA ≥ 0.2) were selected and buried candidates were discarded (at least one residue RSA < 0.2).

**Figure 2:**
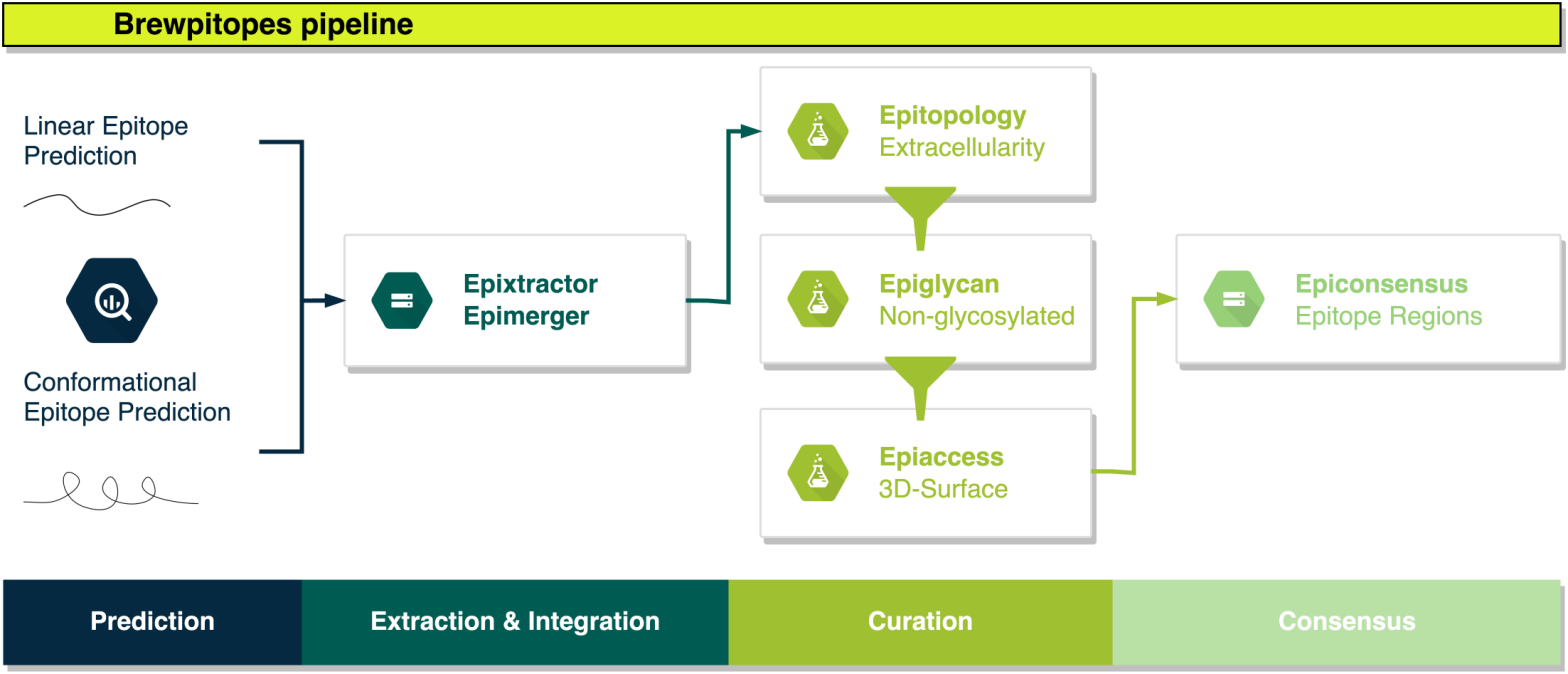
Brewpitopes pipeline. Linear and conformational epitope predictions are performed using Bepipred2.0, ABCpred, and Discotope2.0. Epitope extraction is customized in each tool’s output using Epixtractor. Extracted epitopes are standardized using Epimerger. Subsequently, Brewpitopes implements three *in silico* predictors of biophysical constraints for *in vivo* antibody recognition: subcellular location, glycosylation coverage, and surface accessibility. Protein topology information to determine subcellular location can be uploaded into Brewpitopes using annotated data or via CCTOP predictions (.xml output) using Epitopology. Predicted epitopes located in extracellular regions are selected. Intracellular and transmembrane epitopes are discarded. Glycosylation patterns of target proteins are predicted with Net-N-Glyc and Net-O-Glyc and the output is used by Epiglycan to label all predicted epitopes containing one glycosylated residue as “glycosylated” and candidates not containing glycosylated positions as “non-glycosylated”. Epitope accessibility on the 3D surface of the parental protein structure is computed via *compute_asa.icm* (Molsoft - ICM Browser) and a PDB file obtained from a crystal structure or a computational model. Predicted RSA values are used by Epiaccess to label fully accessible epitopes as “accessible” (all residues RSA ≥ 0.2) and candidates containing at least one buried residue as “buried” (RSA < 0.2). The filtering of the candidate epitopes according to the predicted biophysical constraints (labeled as “extracellular”, “non-glycosylated”, and “accessible”) is performed by Epifilter. Curated candidates predicted by different tools will result in overlapping epitopes that are merged into epitope regions using Epiconsensus.

Brewpitopes was designed as a streamlined pipeline that generates a consensus between linear and conformational epitope predictions and curates them following the *in vivo* antibody recognition constraints (**Figure 1**). To this end, a suite of computational tools was created to integrate the output of different SOTA B-cell epitope predictor and to filter the candidates using predictions of the aforementioned biophysical features (**Figures 1**, **2**).

In Brewpitopes, we included predictions of linear epitopes, which are continual stretches of residues located at the surface of proteins, and predictions of conformational epitopes, which are discontinuous residues recognized by antibodies due to their structural disposition. For both cases, state-of-the-art predictors exist (**Table 1**). To start with, in Brewpitopes, we have predicted linear epitopes using Bepipred2.0 (2) and ABCpred (31) and we have searched for conformational epitopes using Discotope2.0 (8). Once predicted, we have extracted the epitopes using tailored R scripts named Epixtractor and then integrated the results using Epimerger.

Once the predictions are integrated, we propose a set of serial biophysical filters organized in a pipeline. First, since neutralizing antibodies only inspect the external surface of cells or viral particles, we propose that those epitopes predicted in intracellular and transmembrane regions of viral proteins cannot be targets for antibody neutralization (**Figure 1**). Hence, the subcellular location of an epitope is a recognition constraint (47), which our pipeline uses to prioritize epitopes located on extracellular protein regions while discarding those located in intracellular and transmembrane regions. To predict the subcellular location of a protein region, we used protein topology information. For some proteins, the topology is already available at UniProtKB (27); however, for some others, topology is not described. In such cases, the alternative is to predict the topology of the target protein. In Brewpitopes, there is a module to upload experimentally described protein topologies. Complementarily, for undescribed proteins, we used CCTOP to predict their transmembrane, intracellular, and extracellular regions (6). Once we had obtained or predicted the extracellular regions, we labeled the epitopes using Epitopology.

Glycan coverage can limit the surface accessibility of predicted B-cell epitopes that contain glycosylated residues, thus reducing their *in vivo* antibody recognition potential (**Figure 1**) (48). For this reason, our pipeline uses *in silico* tools to predict glycosylated sites on protein sequences. Concretely, we have used NetNglyc1.0 (43) and NetOglyc4.0 (44), for the prediction of N-glycosylations and O-glycosylations, respectively. These methods are based on artificial neural networks trained on glycosylation patterns by which they can predict glycosylation sites *ab initio* given a protein sequence. With this information, Brewpitopes discards all the epitopes that include glycosylated residues using Epiglycan.

As the third filter, we include the accessibility of the epitope within the antigenic protein structure as another antibody recognition constraint (49) (**Figure 1**). Accordingly, our pipeline calculates the relative solvent accessibility (RSA) values of all the residues in the target protein and filters out those epitopes containing at least one buried residue (RSA < 0.2). To compute the RSA values based on crystal structures, we have used Molsoft (45) and the in-house script compute_asa.icm.

The last step of the Brewpitopes pipeline is Epifilter, which uses the annotations of the previous steps to filter out those epitopes predicted as intracellular, glycosylated, or buried. Additionally, a length filter was used to discard epitopes SHORTER than five amino acids in length, which were considered unspecific. Therefore, the final candidates refined using Brewpitopes are extracellular, non-glycosylated, and accessible, properties that enhance the antibody recognition *in vivo*.

The final list of curated epitopes derives from the different tools integrated at the initial step of Brewpitopes. Thus, frequently epitopes with overlapping positions will be encountered. To prevent the prioritization of different but redundant candidates, Brewpitopes merges overlapping epitopes into epitope regions with the aim to generate a consensus between B-cell epitope predictors. Complementarily, the selection of a short sequence length threshold was useful to integrate epitopes predicted by different tools into larger epitope regions. To this end, we designed Epiconsensus, a tool that not only merges overlapping epitopes but also enables the scoring of the merged epitope regions, setting a prioritized order of the initial B-cell epitope predictor scores.

### Bioinformatic validation of Brewpitopes in the proteome of SARS-CoV-2

Brewpitopes can be implemented to any target protein or organism, but due to the pandemic context and the interest in B-cell epitopes and neutralizing antibodies against SARS-CoV-2, to validate the pipeline, we analyzed the proteome of this virus. Within SARS-CoV-2, we specially focused on the S protein due to its importance in vaccine and therapeutic antibody design plus the known role of Spike for immune evasion (50). Our results confirm the neutralizing potential of the S protein but additionally identify other SARS-CoV-2 proteins containing epitopes of interest.

**Figure 3:**
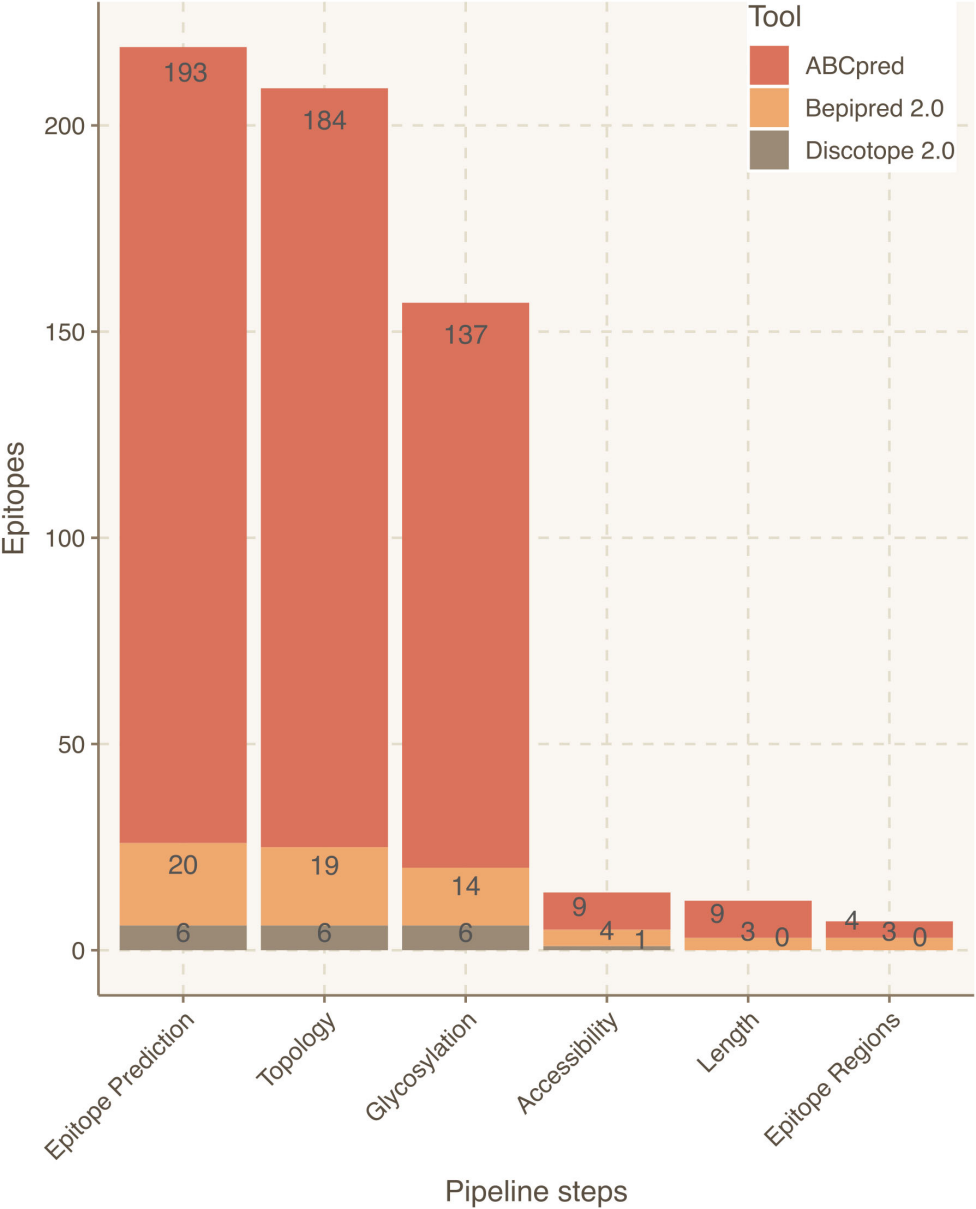
Epitope refinement for SARS-CoV-2 Wuhan S protein. The *x*-axis represents the filtering steps of the pipeline. The *y*-axis displays the number of epitopes refined by each filtering step of Brewpitopes.

**Figure 4:**
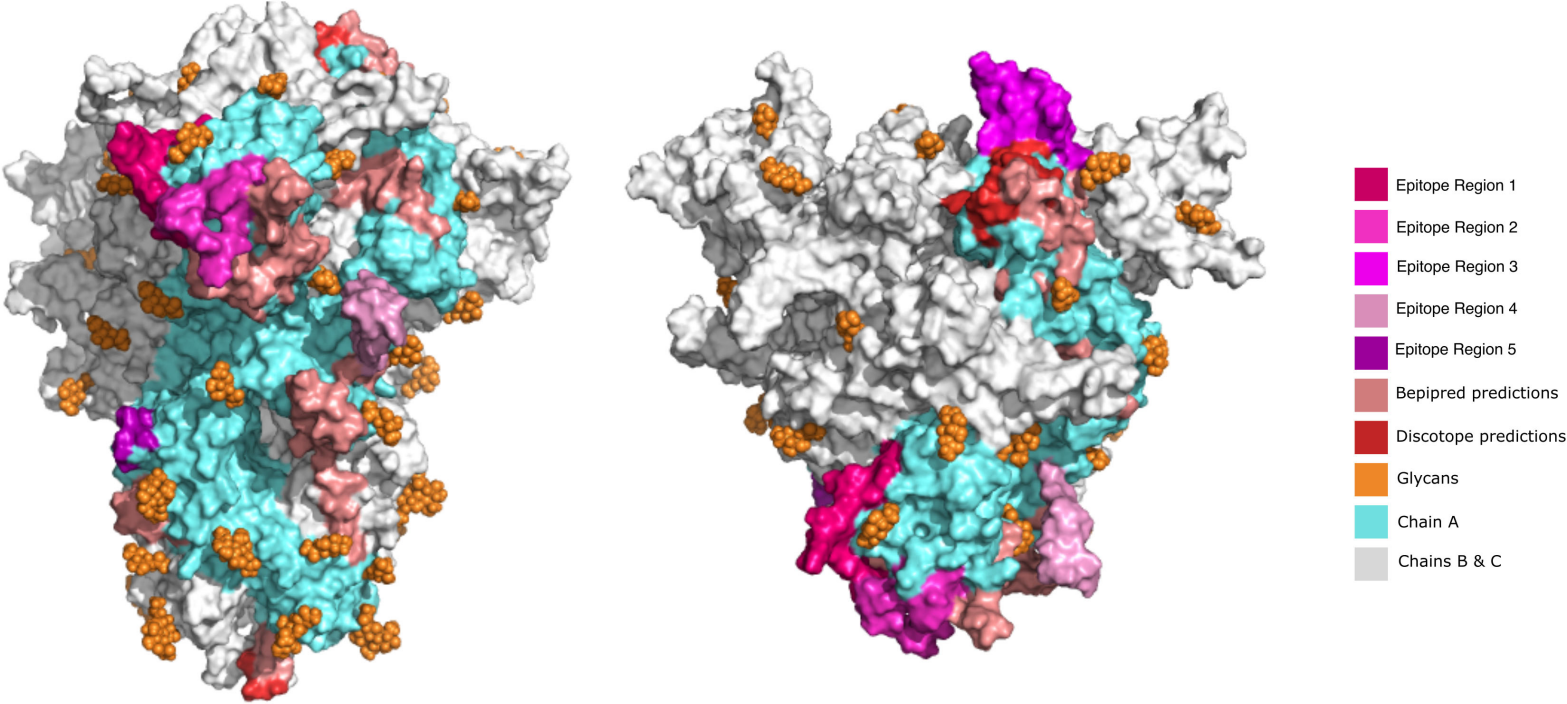
Visualization of predicted epitope location on the 3D structure of SARS-CoV-2 S protein to compare the initially predicted epitopes versus the epitopes refined by Brewpitopes. This representation depicts the shrinkage of the region to be explored and experimentally validated since unrefined predictions represent a much larger surface than the epitopes refined by Brewpitopes. Left: Front view of the S protein 3D structure. Right: Top view. All the epitopes were only labeled on the chain A of the S protein for visualization purposes (blue). The epitope regions 6 and 7 were not displayed because they escaped the limits of the represented structure. Owing to the large number candidates predicted by ABCpred, only the best scored candidates of this software were included in the 3D representation.

Focusing on the S protein, linear epitope predictions resulted in 213 epitopes and structural predictions in 6. Once integrated, 10 epitopes were discarded due to their intraviral location. Next, since it had been established that S protein is heavily glycosylated (26), 52 epitopes were filtered out due to their likelihood to include glycosylated residues. Lastly, 143 epitopes were discarded because they contained at least one residue buried within the 3D structure of the S protein. As a result, 14 epitopes derived from S were curated for optimized antibody recognition (**Figure 3**). Compared to the initial state-of-the-art epitope predictions, our results show that only a 5.5% of the predicted epitopes for the S protein will have high antibody recognition *in vivo* potential due to the recognition constraints analyzed with Brewpitopes (**Figure 4**). Furthermore, to generate a consensus between linear and conformational predictions from different tools, the overlapping epitopes were merged into epitope regions. In the case of S protein, the 14 candidates were merged into seven epitope regions (**Figure 3**).

As an external control, the epitope regions identified in the S protein were cross-validated with the epitopes reported at the IEDB database (51). Notably, the regions identified in our pipeline were all encountered among IEDB annotated epitopes, which confirms the validity of our predictions. However, our epitope regions represented less than 1% of the epitopes for the S protein listed in the IEDB. Compared to the initial output from the computational tools, the final list of prioritized epitopes from our pipeline was enriched fivefold in validated epitopes from IEDB (*p* < 2e-4). This confirms the power of Brewpitopes to refine B-cell epitope computational predictions to a reduced set of epitopes with greater probability for *in vivo* antibody recognition (**Figure 3**).

To extend our proteome-wide analysis of SARS-CoV-2, we used Brewpitopes to search for other epitopes and antigenic viral proteins with antibody recognition potential. Overall, 4/15 of the remaining proteins contained candidate epitopes for neutralizing antibodies (R1AB, R1A, AP3A, and ORF9C) (**Table 2**). The remaining proteins (11/15) did not contain epitopes due to their major intraviral location (NS7A, NS7B, ORF3D, ORF3C, ORF9B, ORF3B, NS8, NS6, M, E, and N) and the absence of predicted epitopes in their short extracellular regions.

**Table 2:** Epitope refinement on SARS-CoV-2 proteome.

Protein	UniProt ID	Predicted Epitopes	Curated Epitopes	Epitope Refinement (%)	Epitopic Regions
**Spike**	P0DTC2	219	12	5.5	7
**E-protein**	P0DTC4	10	0	0	0
**N-protein**	P0DTC9	115	0	0	0
**M-protein**	P0DTC5	22	0	0	0
**R1AB**	P0DTD1	1,111	479	43.1	62
**R1A**	P0DTC1	668	348	52.1	46
**AP3A**	P0DTC3	17	2	11.8	1
**NS6**	P0DTC6	13	0	0	0
**NS7A**	P0DTC7	15	0	0	0
**NS7B**	P0DTD8	2	0	0	0
**NS8**	P0DTC8	14	0	0	0
**ORF3B**	P0DTF1	0	0	0	0
**ORF3C**	P0DTG1	1	0	0	0
**ORF3D**	P0DTG0	12	0	0	0
**ORF9B**	P0DTD2	12	0	0	0
**ORF9C**	P0DTD3	9	4	44.44	2

Predicted epitopes correspond to the number of epitopes obtained using individual linear and structural predictors. Curated epitopes refer to refined epitopes obtained using Brewpitopes. Epitope refinement is the percentage of curated epitopes over the initial number of predicted epitopes obtained using individual state-of-the-art tools. Epitope regions result from the integration of overlapping predictions by different tools.

Within the proteins that contained curated epitopes, R1AB and R1A stood out, including 479 and 348 epitopes, respectively. The large numbers of epitopes predicted in these proteins is mainly explained by their long sequences, 7,096 and 4,405 amino acids, respectively. Remarkably, R1A corresponds to the N-terminal region of R1AB explaining the high degree of shared predictions. R1AB is a complex polyprotein cleaved into 15 chains. In this analysis, all the chains were analyzed together using the standard R1AB UniProt sequence. On the other hand, we could also identify epitopes located in shorter proteins as ORF9C and AP3A. Accordingly, these presented a lower number of predicted candidates. In terms of epitope regions, R1AB contains 62 regions; R1A, 46 regions; ORF9C, 2 regions; and AP3A, 1 region. Altogether, these results corroborate that four SARS-CoV-2 proteins other than S have at least one candidate epitope region with *in vivo* antibody recognition potential.

### Analysis of epitope conservation in the S protein of variants of concern

**Figure 5:**
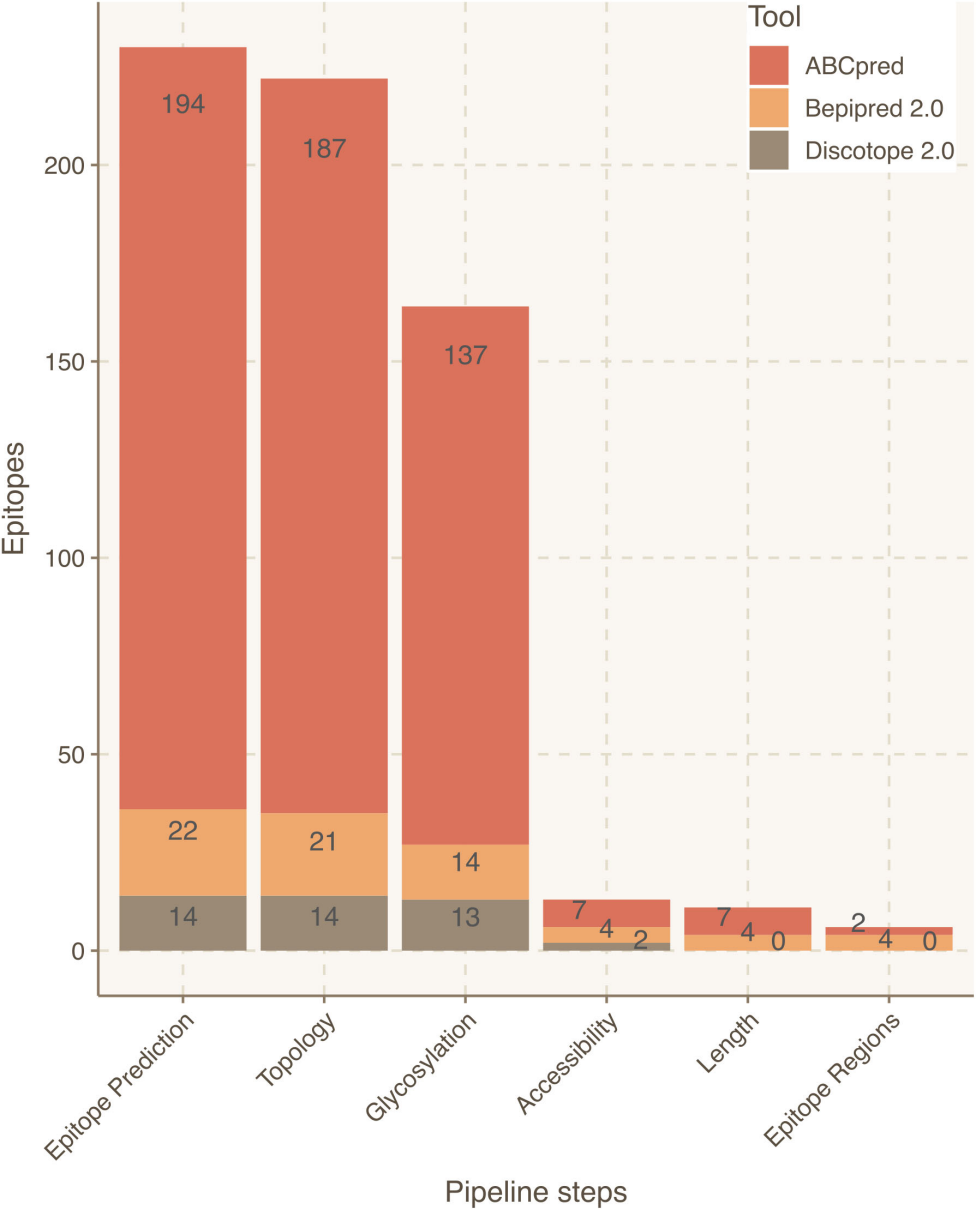
Epitope refinement for the S protein of the Omicron variant. The *x*-axis represents the steps of the Brewpitopes pipeline and the *y-*axis denotes the number of epitopes selected by each filtering step of Brewpitopes (**Figure 2**). Omicron’s epitope yield obtained with Brewpitopes (six epitope regions) is lower than Wuhan WT’s yield (seven epitope regions).

We studied the effect of mutations accumulated in the S protein of the VOCs (Alpha, Beta, Delta, Gamma, and Omicron) of SARS-CoV-2 in the development of immune escape mechanisms implementing Brewpitopes on the S protein sequences of the different variants (**Table S1**; **Figure 5**). We generated tailored FASTA files including the mutations of each variant and we retrieved the structures from PDB when available. For the Omicron variant, we modeled its structure using Modeller (30). Once we had run Brewpitopes, we compared the final number of epitopes with neutralizing potential identified in each variant with the epitopes generated by our analysis of the Wuhan S protein, considered the wild type. Concretely, we aimed at identifying epitope losses due to the presence of mutations, the appearance of new glycosylation sites and structures changed, leading to new buried positions. Additionally, we accounted for newly predicted epitopes generated by unique mutations of each variant. To compare epitope regions in WT versus those of the VOCs, the length of these epitope regions was added and divided by the total length of the S protein to obtain a protein-wide epitope coverage metric. In other words, this metric is the fraction of the protein sequence that is covered by predicted epitopes. This analysis predicted an epitope coverage of 9.43% for the WT S variant.

To visualize the accumulation of mutations in the VOC’s S protein, we calculated the intersections of shared mutations between variants (**Table S1**; **Figure S1**). Accordingly, the UpSet plot shows how the Omicron variant accumulates the largest number of mutations (4), of which 28 are exclusive. Gamma accumulates eight unique mutations; Delta, seven mutations; Beta, six mutations; and Alpha, four mutations. Also, the degree of shared mutations between variants is low, with Alpha and Omicron being the variants that share more mutations, with four. The other VOC’s pairs share a single mutation while the intersection of all variants also points to a single foundational mutation. This high diversity in the mutations accumulated in S protein across variants points towards separate evolutionary paths. This phenomenon can derive into variant-specific immune evasion mechanisms such as decreased antibody recognition. The fact that Omicron accumulates more than three times more mutations at S than the remaining VOCs indicates a greater potential for epitope disruption.

The accumulation of more variant-specific mutations in the S protein than shared mutations (**Figure S1**; **Table S1**) implies a potential development of specific epitope landscape in each variant (**Tables 3**, **4**). Additionally, these variant landscapes are likely to differ from the patterns observed in the WT Wuhan variant. Considering epitope region conservation against the wild-type virus, the Alpha variant loses ER7; the Beta variant loses ER4 and ER7 but gains an epitope region at 828–845; the Gamma variant loses ER2, ER3, and ER4; the Delta variant loses ER3, ER4, and ER6 but gains ER1, ER5, and ER8; and the Omicron variant loses ER2, ER3, and ER4 partially and ER7 entirely (**Table 4**; **Figure 5**).

**Table 3:** Epitope refinement on S protein in Wuhan and Alpha, Beta, Delta, Gamma, and Omicron variants.

Variant	ID	Predicted Epitopes	Curated Epitopes	Epitopic Regions	Epitope Refinement (%)	Epitope Conservation (%)	Epitopic Region Conservation (%)
Wuhan-2	WT	219	12	7	5.5	100	100
Alpha	B.1.1.7	206	13	6	6.3	108.3	85.7
Beta	B.1.351	225	11	6	4.9	91.7	85.7
Delta	P.1	213	15	7	7	125	100
Gamma	B.1.617.2	214	6	5	2.8	50	71.4
Omicron	B.1.1.529	230	11	6	4.8	91.7	85.7

Predicted epitopes correspond to the number of epitopes obtained using individual linear and structural predictors. Curated epitopes refer to refined epitopes obtained using Brewpitopes. Epitope refinement is the percentage of curated epitopes over the initial number of predicted epitopes obtained using individual state-of-the-art tools. Epitope regions result from the integration of overlapping predictions by different tools.

Epitope conservation refers to the percentage of refined epitopes shared between each variant and the WT S protein. Epitope region conservation refers to the percentage of epitope regions shared between each variant and the WT S protein.

**Table 4:** Epitope regions identified in the WT S protein using Brewpitopes compared to the epitope regions of the variants of concern.

Variant	Wuhan_2	Alpha	Mutations	Glycosilations	Buried
**Epitope Region 1**	NA	NA	NA	NA	NA
**Epitope Region 2**	168-FEYVSQPFLMDLEGKQGN-185	164-TFEYVSQPFLMDLEGKQGNFK-184	NA	NA	NA
**Epitope Region 3**	244-LHRSYLTPGDSSSGWTA-260	248-PGDSSSGWT-256	NA	NA	NA
**Epitope Region 4**	470-TEIYQAGSTPCNGVEGFNCYFP-491	NA	NA	NA	472, 475, 487, 488, 491
**Epitope Region 5**	NA	NA	NA	NA	NA
**Epitope Region 6**	621-PVAIHADQLTPTWRVYSTGS-640	620-AIHADQLTPTWRVYSTGSNVFQT-642	NA	NA	NA
**Epitope Region 7**	809-PSKPS-813	NA	NA	NA	NA
**Epitope Region 8**	NA	828-AGFIKQYGDCLGDIAARD-845	NA	NA	NA
**Epitope Region 9**	1155-YFKNHTSPDVDLGDISGINASV-1176	1152-YFKNHTSPDVDLGDISGINASVVNIQKE-1179	NA	NA	NA
**Epitope Region 10**	1195-ESLIDLQELGKYEQYI-1210	1192-ESLIDLQELGKYEQYI-1207	NA	NA	NA
Variant	Wuhan_2	Beta	Mutations	Glycosilation	Buried
**Epitope Region 1**	NA	NA	NA	NA	
**Epitope Region 2**	168-FEYVSQPFLMDLEGKQGN-185	176-LMDLEGKQGNFK-187	NA	NA	168
**Epitope Region 3**	244-LHRSYLTPGDSSSGWTA-260	249-GDSSSGW-255	NA	NA	241*
**Epitope Region 4**	470-TEIYQAGSTPCNGVEGFNCYFP-491	NA	E484K	NA	472, 475, 480, 487, 488, 491
**Epitope Region 5**	NA	NA	NA	NA	NA
**Epitope Region 6**	621-PVAIHADQLTPTWRVYSTGS-640	620-AIHADQLTPTWRVYSTGSNVFQT-642	NA	NA	NA
**Epitope Region 7**	809-PSKPS-813	NA	NA	NA	806*
**Epitope Region 8**	NA	828-AGFIKQYGDCLGDIAARD-845	NA	NA	NA
**Epitope Region 9**	1155-YFKNHTSPDVDLGDISGINASV-1176	1152-YFKNHTSPDVDLGDISGINASVVNIQKE-1179	NA	NA	NA
**Epitope Region 10**	1195-ESLIDLQELGKYEQYI-1210	1192-ESLIDLQELGKYEQYI-1207	NA	NA	NA
Variant	Wuhan_2	Gamma	Mutations	Glycosilations	Buried
**Epitope Region 1**	NA	NA	L18F, T20N	17	NA
**Epitope Region 2**	168-FEYVSQPFLMDLEGKQGN-185	NA	NA	NA	168
**Epitope Region 3**	244-LHRSYLTPGDSSSGWTA-260	NA	NA	NA	244, 246, 258
**Epitope Region 4**	470-TEIYQAGSTPCNGVEGFNCYFP-491	NA	E484K	NA	473, 475, 476, 487, 488, 489, 491
**Epitope Region 5**	NA	NA	NA	NA	NA
**Epitope Region 6**	621-PVAIHADQLTPTWRVYSTGS-640	621-PVAIHADQLTPTWRVYSTGS-640	NA	NA	NA
**Epitope Region 7**	809-PSKPS-813	809-PSKPS-813	NA	NA	NA
**Epitope Region 8**	NA	NA	NA	NA	NA
**Epitope Region 9**	1155-YFKNHTSPDVDLGDISGINASV-1176	1141-LQPELD-1146//1155-YFKNHTSPDVDLGDISGINASF-1176	NA	NA	NA
**Epitope Region 10**	1195-ESLIDLQELGKYEQYI-1210	1195-ESLIDLQELGKYEQYI-1210	NA	NA	NA
Variant	Wuhan_2	Delta	Mutations	Glycosilations	Buried
**Epitope Region 1**	NA	14-QCVNLRTRTQ-23	T19R	NA	NA
**Epitope Region 2**	168-FEYVSQPFLMDLEGKQGN-185	NA	NA	NA	173
**Epitope Region 3**	244-LHRSYLTPGDSSSGWTA-260	243-HRSYLTPGDSSSGWTA-258	NA	NA	NA
**Epitope Region 4**	470-TEIYQAGSTPCNGVEGFNCYFP-491	NA	T478K	NA	478
**Epitope Region 5**	NA	496-QPTNG-500	NA	NA	NA
**Epitope Region 6**	621-PVAIHADQLTPTWRVYSTGS-640	NA	NA	NA	631
**Epitope Region 7**	809-PSKPS-813	807-PSKPS-811	NA	NA	NA
**Epitope Region 8**	NA	827-ADAGFIKQYGDCLGDIAA-844	NA	NA	NA
**Epitope Region 9**	1155-YFKNHTSPDVDLGDISGINASV-1176	1136-YDPLQPELDSFKEELDKYFKNHTSPDVDLGDISGINASVVNIQKEIDRLNEVAKN-1190	NA	NA	NA
**Epitope Region 10**	1195-ESLIDLQELGKYEQYI-1210	1193-ESLIDLQELGKYEQYIKWPW-1212	NA	NA	NA
Variant	Wuhan_2	Omicron	Muts	Glycosilations	Buried
**Epitope Region 1**	NA	NA	NA	N17	NA
**Epitope Region 2**	168-FEYVSQPFLMDLEGKQGN-185	178-QGNFK-182	NA	NA	172
**Epitope Region 3**	244-LHRSYLTPGDSSSGWTA-260	248-PGDSSSGWT-256	NA	NA	243*
**Epitope Region 4**	470-TEIYQAGSTPCNGVEGFNCYFP-491	467-TEIYQAGNKPCNGVAGFNCYFPL-489	S477N, T478K, E484A	NA	491
**Epitope Region 5**	NA	NA	G496S, Q498R	NA	495, 497
**Epitope Region 6**	621-PVAIHADQLTPTWRVYSTGS-640	627-TPTWRVYSTGSNVFQT-642	NA	NA	620*
**Epitope Region 7**	809-PSKPS-813	NA	NA	NA	815, 816*
**Epitope Region 8**	NA	NA	NA	NA	(…)
**Epitope Region 9**	1155-YFKNHTSPDVDLGDISGINASV-1176	1152-YFKNHTSPDVDLGDISGINASVVNIQKE-1179	NA	NA	NA
**Epitope Region 10**	1195-ESLIDLQELGKYEQYI-1210	1192-ESLIDLQELGKYEQYI-1207	NA	NA	NA

“Mutations”, “Glycosylations”, and “Buried” refer to the residues that cause the disruption of epitope regions in each viral variant.

In terms of epitope coverage, the major loss is prediction on Gamma (4%) and Omicron (2%) variants while Alpha and Beta loss is less than 1.5%. Differently, Delta gains 0.5% in epitope coverage in respect to WT due to the prediction of a large epitope. The differences in variant epitope landscape can be attributed to partial losses in antibody recognition. However, using Brewpitopes, a core of epitope regions conserved across variants could be identified (**Table 5**).

**Table 5:** Epitope coverage of the WT S protein versus variants of concern.

Variant	Epitope Coverage (%)
Wuhan_2	9.43
Alpha	9.03
Beta	8.17
Delta	10.13
Gamma	5.42
Omicron	7.62

Epitope coverage is the percentage of the total protein sequence (the S protein) that is covered by curated epitope regions predicted using Brewpitopes. It estimates the antigenicity potential of a protein. The loss of epitope coverage in variants of concern is a proxy to estimate their immune escape potential due to the loss of in vivo antibody neutralization.

## Discussion

*In vivo* antibody recognition is constrained by molecular features not frequently integrated in state-of-the-art B-cell epitope predictors. These include extracellular location of the epitope, absence of glycosylation coverage, and surface accessibility on the parental protein (**Table 1**). In Brewpitopes, we have implemented these features as filters to refine bioinformatic B-cell epitope predictions. Thus, Brewpitopes optimizes *in vivo* antibody recognition properties of predicted epitopes. The proteome-wide SARS-CoV-2 analysis demonstrates the obtainment of a refined set of epitopes with neutralizing potential in S protein and its conservation in VOCs (Alpha, Beta, Delta, Gamma, and Omicron). Additionally, we identified four proteins with candidate epitope regions for neutralization studies. As exemplified in this study, Brewpitopes is a ready-to-use tool to enhance the accuracy and response rates of bioinformatic B-cell epitope predictions for future public health emergencies such as the appearance of vaccine-resistant SARS-CoV-2 variants and other pathogenic threads.

Profiling of the B-cell epitope landscape in SARS-CoV-2 has been a research-intensive topic since the start of the COVID-19 pandemic for its implications in vaccine and therapeutic antibody development (**Table 1**) (14–22, 52, 53). However, none of the proposed strategies jointly integrates the prediction of subcellular location, glycosylation status, or 3D accessibility of the epitope as factors influencing antibody recognition. For this reason, Brewpitopes is a first-in-class pipeline thanks to a streamlined implementation of *in silico* predictors of biophysical constraints. Furthermore, the available methods can only predict linear or conformational epitopes separately, whereas with Brewpitopes, we propose an integration of both types of predictions into linear epitope regions using the Epiconsensus tool.

The filters implemented in Brewpitopes are based on computational predictions, such as CCTOP for subcellular location of protein regions or Net-N-glyc and Net-O-glyc for glycosylations. The usage of bioinformatic tools expands the applicability of Brewpitopes enabling *ab initio* predictions on the proteome of understudied organisms or new pathogens. These tools preclude the requirement of previous protein topology, glycosylation, and accessibility of experimental determinations. Thus, Brewpitopes can be implemented rapidly and without large resource requirements. However, relying on bioinformatic predictions inevitably implies at least a minimal degree of false positives and false negatives among the curated and discarded candidates.

In the case of glycosylation predictions, the dynamics of this type of PTM or its effects on neighboring epitopes cannot be assessed *in silico* using a sequence-based approach as Brewpitopes. In terms of structural accessibility, many candidates predicted by individual tools used in this study contained buried residues. This can limit the recognition of the candidates as compared to fully accessible epitopes (47). To minimize this effect, in Brewpitopes, we discard all epitopes containing a single buried residue (RSA <0.2). This criterion is the most stringent filter of the pipeline. In the case of S protein, it downsized the number of candidates from 137 to 14 (**Figure 3**; **Table 2**). As expected, after the implementation of this stringent filter, a proportion of epitopes discarded may still have antigenic activity. Still, since the objective of the pipeline is to obtain the greatest immunogenicity enrichment in the refined candidates; we consider that this filter strongly serves this purpose. Complementarily, accessibility predictions depend on optimal structural resolution, which is difficult to obtain for highly flexible protein regions. To circumvent this, we labeled these regions as unmodeled, but due to their high flexibility, these were included as exposed regions and epitopes predicted within these passed the accessibility filter.

In terms of software flexibility, Brewpitopes is built upon Discotope2.0, and Bepipred2.0, which, during the pipeline development and SARS-CoV-2 analysis, were considered state of the art by the IEDB analysis resource tool (51). ABCpred was also included in the analysis, but it can no longer be considered a cutting-edge method. Accordingly, Brewpitopes succeeds in discarding a major quantity of candidates predicted by this tool. In addition, Brewpitopes’ design flexibility enables a straightforward integration of new state-of-the-art methods and can be easily maintained to keep up with the fast evolution pace of the field.

While Brewpitopes can be applied to any protein or organism, given the wealth of SARS-CoV-2 data and biomedical interest, we focused on the analysis of this virus. We performed a proteome-wide analysis of the epitope landscape in SARS-CoV-2 to obtain a curated list of epitopes with neutralizing potential. To study the immune evasion mechanisms by SARS-CoV-2, we predicted the epitope profiles of WT S protein and we assessed how these were affected by variant-specific mutations. This comparison led to the discovery of six epitope regions conserved across variants, which could explain the conserved protection of vaccinated patients against new variants (54). In this line, the restrictive nature of Brewpitopes’ filtering criteria led to a significant reduction of predicted epitopes on the S protein to be validated. This study serves as an example of the value of the pipeline in terms of experimental resource optimization.

The identification of potentially neutralizing epitopes in R1AB, R1A, AP3A, and ORF9C highlights the importance of studying proteome regions with low variability. Despite the fact that these proteins are not considered key for viral survival and cellular entry, the presence of extracellular regions accessible for antibody recognition supports their neutralizing potential. The restricted viral evolution of these proteins can limit the advantage of variants in terms of antigen drift and immune escape while leading to greater vaccine protection rates.

Despite losses in epitope coverage observed in S protein variants, Brewpitopes could identify several epitope regions shared across variants. This finding has beneficial implications for vaccine efficacy versus new VOCs. Brewpitopes reported a lower epitope coverage loss for Omicron than for the Gamma variant. The epitope coverage loss predicted in Omicron versus Wuhan could partially explain the large loss of neutralization against this variant reported by previous studies (55). Discordances between neutralization studies (55) and the results of Brewpitopes can be explained by relevant differences between *in vitro* and *in silico* methods. As aforementioned, Brewpitopes’ stringency could discard a proportion of truly antigenic epitopes and thus underrepresent the neutralization loss observed in Omicron.

Brewpitopes is a pipeline that refines bioinformatic B-cell epitope predictions straightforwardly for use against any target protein or organism’s proteome. The integration of multiple state-of-the-art B-cell epitope algorithms coupled with the addition of *ab initio* predictions of important features for *in vivo* antibody recognition is a relevant advantage over existing pipelines and individual predictors. Furthermore, implementing Brewpitopes to the proteome of SARS-CoV-2 Wuhan WT variant versus VOCs, we have identified an epitope core in S protein conserved across variants and new antigenic regions in four SARS-CoV-2 proteins less prone to immune escape due to lower immune pressure and antigenic drift rates.

In conclusion, Brewpitopes is a streamlined pipeline that assesses biophysical properties not accounted for in state-of-the-art B-cell epitope predictors. The usage of *in silico* predictors of subcellular location, glycosylation status, and surface accessibility has been demonstrated as crucial to enrich the neutralization potential of predicted epitopes in SARS-CoV-2.

## Data availability statement

The original contributions presented in the study are included in the article/**Supplementary Material**. Further inquiries can be directed to the corresponding authors.

## Author contributions

RFD: Investigation, Methodology, Writing – original draft, visualization, conceptualization and investigation. RL-A: Investigation, Writing – original draft. EPP: Conceptualization,Supervision, Writing – review & editing, funding acquisition, methodology, validation and visualization. AT: Funding acquisition, Supervision, Writing – review & editing. LF-B: Funding acquisition, Investigation, Methodology, Project administration, Resources, Supervision, Validation, Visualization, Writing – original draft.

## Supplementary material

Supplementary Figure 1 Mutations accumulated in the protein S of the Variants of Concern Alpha, Beta, Delta, Gamma and Omicron. Representation of unique and shared mutations of each variant. Total mutations per each variant are displayed in the lower barplot. The accumulation of mutations in the S protein of viral variants can be linked to a greater potential of immune escape due to the potential disruption of epitopes caused by changes in the sequence. Omicron stands out accumulating the 3 times more mutations than other variants.

Supplementary Table 1 Amino acid changes and sequence position of mutations in the S protein of variants of concern.

Supplementary Table 2 Comparison of glycosylation sites in S protein determined by MS or predicted using computational tools. Experimental sources include mass spectrometry glycosylation studies. *In silico* glycosylation sites were predicted using computational tools (Net-N-Glyc and Net-O-Glyc).
