# Supplementary figures and images for: Brewpitopes: a pipeline to refine B-cell epitope predictions during public health emergencies
Source: Front Immunol. 2023 Dec 6;14:1278534. doi: 10.3389/fimmu.2023.1278534 (PMC10730938; doi:10.3389/fimmu.2023.1278534)

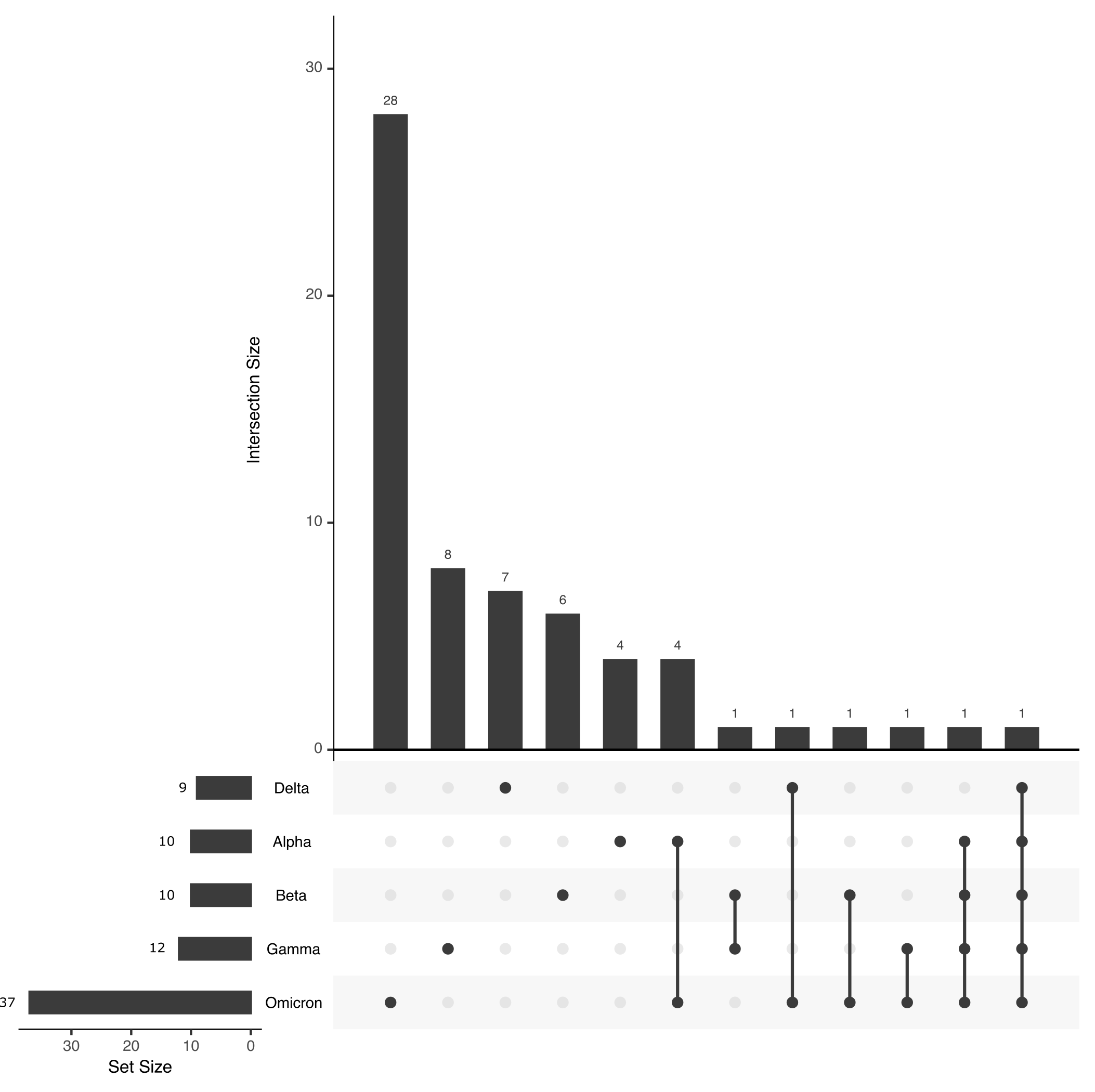

Supplement: Supplementary Figure 1 — Mutations accumulated in the protein S of the Variants of Concern Alpha, Beta, Delta, Gamma and Omicron. Representation of unique and shared mutations of each variant. Total mutations per each variant are displayed in the lower barplot. The accumulation of mutations in the S protein of viral variants can be linked to a greater potential of immune escape due to the potential disruption of epitopes caused by changes in the sequence. Omicron stands out accumulating the 3 times more mutations than other variants. [file Image_1.tiff]
